# Insomnia in adults with cystic fibrosis: strong association with anxiety/depression and impaired quality of life

**DOI:** 10.1186/s12890-021-01473-y

**Published:** 2021-04-01

**Authors:** Pauline Mulette, Bruno Ravoninjatovo, Camille Guguen, Coralie Barbe, Julien Ancel, Sandra Dury, Antoine Dumazet, Dominique Perdu, Jeanne-Marie Perotin, Thomas Guillard, François Lebargy, Gaëtan Deslee, Claire Launois

**Affiliations:** 1 Service Des Maladies Respiratoires, CHU Reims, 45, Rue Cognacq Jay Reims Cedex, 51092 Reims, France; 2 Inserm UMR-S 1250 “Pathologies Pulmonaires et Plasticité Cellulaire”, Reims, France; 3 Département de Méthodologie, CHU Reims, Reims, France; 4 EA 4683 Université de Médecine et de Pharmacie, Reims, France; 5 Laboratoire de Bactériologie-Virologie-Hygiène-Parasitologie-Mycologie, CHU Reims, Reims, France

**Keywords:** Cystic fibrosis, Insomnia, Sleep, Anxiety-depression, Quality of life

## Abstract

**Background:**

While sleep disruption is a common complaint among children with cystic fibrosis (CF), only a few studies have investigated insomnia in adults. The aim of this study was to identify factors associated with insomnia in clinically stable adult CF patients.

**Methods:**

Twenty-eight CF patients (18M/10F), with a median age of 27 (22–34) (median (interquartile range) years and a median of forced expiratory volume in one second of 72 (39–93) % predicted completed questionnaires on insomnia (Insomnia Severity Index, ISI), sleep quality (PSQI), daytime sleepiness (Epworth), restless legs syndrome (IRLS), pain (NRS), anxiety/depression (HAD) and quality of life (CFQ-R 14+). Respiratory assessment data, including symptoms, sputum analysis, arterial blood gases, 6-min walking test, pulmonary function tests and polysomnographic variables, were also analyzed.

**Results:**

Forty-three percent of patients were insomniac (ISI > 7). Compared with non-insomniac patients (ISI ≤ 7), insomniac patients had more severely impaired quality of life and a higher HAD score: median anxiety score of 9 (8–11) vs 4 (3–6) (*p* < 0.0001), median depression score of 7 (5–10) vs 1 (1–4) (*p* < 0.001), with a positive correlation between ISI and HAD anxiety/depression scores (r = 0.702/r = 0.701, respectively, *p* < 0.0001). Insomnia was also associated with mMRC dyspnea scale ≥ 2, restless legs syndrome, pain and lower SpO_2_ during sleep.

**Conclusions:**

The strong association between insomnia, impaired quality of life and increased HAD score should prompt physicians to be particularly attentive to the management of anxiety and depression in adult CF patients with insomnia.

*Trial registration*: On clinicaltrials.gov (NCT02924818, date of registration: October 5, 2016).

## Background

Cystic fibrosis (CF) is currently the most common lethal genetic disease. Registry-derived population estimates suggest in excess of 72 000 people are living with CF worldwide [1], with an expected large increase in CF adults during the next decade [2]. CF is caused by mutations in the gene encoding the cystic fibrosis transmembrane conductance regulator protein, an anion channel that regulates the activity of other ion transporters and governs the hydration and viscoelastic properties of mucus in several epithelial tissues [3]. As a consequence of abnormal mucus secretions, dysfunction in organ systems including lungs, gastrointestinal tract, liver, male reproductive tract and pancreas occurs. It results in many symptoms especially cough, nasal obstruction and abdominal pain [4]. Despite improvements in life expectancy, many patients with CF experience a considerable daily symptom.

Sleep disruption is also a common complaint in CF children [5] that contributes to impaired daytime functioning and quality of life [6]. In adults, CF-related sleep quality has been the primary focus of a small number of studies [7–12]. As the most common cause of both morbidity and mortality in CF patients is respiratory disease [13], most of these studies focused on the associations between sleep quality and impaired lung function. Milross et al. demonstrated associations between poorer sleep quality, defined as Pittsburgh Sleep Quality Index (PSQI) score > 5, and more severely impaired lung function and gas exchange in adult patients with CF [10]. The mean arterial oxygen level during sleep was also associated with reduced sleep efficiency in adult CF patients with severe lung disease [9].

In addition to lung function, other factors are likely to impair sleep quality in CF adult patients such as night-time symptoms (cough [14], pain [15]), restless legs syndrome (RLS) [16, 17], anxiety and depression [12, 18], sleep-related breathing disorders [19, 20], acute pulmonary exacerbation [21] or unhealthy lifestyle.

The insomnia severity index (ISI) is an effective brief instrument to assess insomnia, which consists of seven self-reported items [22]. It has been shown to be reliable and has been validated against several other sleep questionnaires, including the PSQI [23].

The objective of this study was to identify factors associated with insomnia, as assessed by ISI, in clinically stable CF adults and to analyze relationships between insomnia and quality of life.

## Methods

Adult CF patients were recruited from the Reims University Hospital Department of pulmonary medicine (France) and were included in the Research and Innovation in Chronic Inflammatory Respiratory Diseases (RINNOPARI) cohort. This study was performed in accordance with the declaration of Helsinki and approved by the Ethics Committee (*Comité de Protection des Personnes*—Dijon EST I, No. 2016-A00242-49) and was registered on clinicaltrials.gov (NCT02924818, date of registration: October 5, 2016). Adult CF patients referred to our center between January 2017 and June 2019 were considered for inclusion in this study. All patients received detailed information about the methods used and gave their written consent.

The RINNOPARI project is a general project designed to study clinical, laboratory, functional, morphological, histological and microbiological characteristics in patients with chronic inflammatory lung diseases, including CF.

### Patient selection

Inclusion criteria were age older than 18 years, a diagnosis of CF confirmed by sweat tests and/or genetic analysis, and absence of long-term oxygen, bi-level positive airway pressure therapy or lung transplantation. The exclusion criterion was an ongoing or recent (i.e. within the last 4 weeks prior to study recruitment) medical condition, including pulmonary exacerbations and pregnancy.

### Patient demographic and clinical characteristics

Demographic data (age, sex), body mass index (BMI), comorbidities, smoking status, alcohol, coffee, tea, energy drink consumption and treatments were systematically recorded. Patients filled in self-administered questionnaires on cough (*Cough And Sputum Assessment Questionnaire: CASA-Q*), dyspnea (*modified Medical Research Council dyspnea scale: mMRC*), pain (*Numeric Rating Scale: NRS*), insomnia (*Insomnia Severity Index scale*), sleep quality (*Pittsburgh Sleep Quality Index*), daytime sleepiness (*Epworth Sleepiness scale: ES*), snoring (*Berlin Questionnaire)*, circadian rhythm (*Morningness-Eveningness Questionnaire: MEQ*), anxiety and depression (*Hospital Anxiety and Depression Scale: HAD*). Patients were asked about the presence or absence of restless legs syndrome according to the International Restless Legs Syndrome consensus criteria [24], the severity of which was assessed by the *International Restless Legs Syndrome scale (IRLS).* The patients' quality of life was also assessed by the *Cystic Fibrosis Questionnaire-Revised (CFQ-R 14+)*. Information regarding pathogens detected in sputum, especially the presence of *Pseudomonas aeruginosa*, *Burkholderia cepacia*, *Stenotrophomonas maltophilia* and *Achromobacter xylosoxidans* was collected.

#### Cough And Sputum Assessment Questionnaire

The CASA-Q is a self-administered questionnaire assessing cough and sputum based on their frequency, severity, and impact on daily activities over the previous 7 days. It comprises four domains: cough symptoms, cough impact, sputum symptoms, and sputum impact. Each domain comprises three to eight items, each of which is scored according to five categories from “never” to “always” for frequency and from “not at all” to “a lot/extremely” for severity. For each domain, the items are summed and rescaled to obtain a score ranging from 0 to 100, with higher scores associated with fewer symptoms or less impact [25].

#### Modified Medical Research Council Dyspnea Scale

The mMRC scale consists of five statements that almost entirely describe the range of dyspnea from none (grade 0) to almost complete incapacity (grade 4) [26]. Significance thresholds of 1 and 2 has been used to categorized patients according to their dyspnea.

#### Numeric Rating Scale for pain

The NRS was used to measure pain intensity, with scores ranging between 0 and 10 (0: no pain, 10: maximum pain) [27].

#### Insomnia Severity Index

The ISI is a self-reporting instrument measuring the patient’s perception of his/her insomnia. It comprises seven items assessing the severity of sleep-onset and sleep maintenance difficulties (both nocturnal and early morning awakenings), satisfaction with current sleep pattern, interference with daily functioning, impairment attributed to sleep problem, and degree of concern caused by the sleep problem. Each item is rated on a 0–4 scale and the total score ranges from 0 to 28. A score between 0 and 7 is considered not to be clinically significant, a score of 8–14 indicates mild insomnia, a score of 15–21 indicates moderate insomnia and a score of 22–28 indicates severe insomnia [22].

#### Pittsburgh Sleep Quality Index

The PSQI is a self-rated questionnaire, which assesses sleep quality and disturbances over a 1-month time interval. Nineteen individual items generate seven component scores: subjective sleep quality, sleep latency, sleep duration, habitual sleep efficiency, sleep disturbances, use of sleeping medication and daytime dysfunction. The sum of the component scores yields a global score from 0 to 21. A global PSQI score > 5 indicates impaired sleep quality [28].

#### Epworth Sleepiness Scale

The ES is a self-administered questionnaire, in which an individual rates the likelihood of falling asleep under various circumstances in daily life. The scale ranges from 0 to 24. Excessive daytime sleepiness is defined by a score ≥ 11 [29].

#### Berlin Questionnaire

The Berlin Questionnaire includes five items on snoring (category 1), three items on daytime somnolence (category 2), and one item on the history of hypertension and/or BMI > 30 kg/m^2^ (category 3). Patients are considered to be at risk for obstructive sleep apnea when at least two categories are positive [30].

#### Morningness-Eveningness Questionnaire

The MEQ is used to determine the patient's circadian rhythm. It consists of 19 mixed-format questions regarding the time individuals get up and go to bed, preferred times for physical and mental activity, and subjective alertness. The MEQ score ranges from 16 to 86, with scores above 58 classifying individuals as morning-type and scores below 41 as evening-type [31].

#### International Restless Legs syndrome scale

Restless Legs Syndrome (RLS) was diagnosed when the patient satisfied the International RLS consensus criteria [24]. The severity of RLS was evaluated using the IRLS, which is composed of 10 questions. Each question can be classified according to severity, as follows: none (0 point), mild (1 point), moderate (2 points), severe (3 points), and very severe (4 points). The total score ranges from 0 to 40. Participants with IRLS scores < 10 were categorized as mild, 11–20 as moderate, 21–30 as severe, and ≥ 31 as very severe RLS [32].

#### Hospital Anxiety and Depression scale

The Hospital Anxiety and Depression scale is a self-administered rating scale specifically designed for patients with physical illness. It consists of 14 items: seven items relating to depression and seven items relating to anxiety with cut-off points for severity. For each domain (A or D), scores of 0–7 indicate no disorders, 8–10 indicate suspected disorders, and 11–21 indicate confirmed disorders [33].

#### Cystic Fibrosis Questionnaire-Revised

The CFQ-R 14+consists of 50 items structured in 12 domains that, in turn, are divided into 6 domains that assess general aspects of health-related quality of life: physical functioning, role limitations, vitality, health perceptions, emotional state and social functioning and 6 domains that address specific aspects of CF: body image, eating problems, treatment burden, weight problems, respiratory symptoms and digestive symptoms. Scores range from 0 to 100, and higher scores correspond to better quality of life [34].

### Pulmonary function

Pulmonary function tests (PFTs) were performed according to the American Thoracic Society/European Thoracic Society guidelines [35] (BodyBox 5500 Medisoft Sorinnes, Belgium).

The 6-min walking test (6MWT) was performed according to the American Thoracic Society guidelines (ATS 2002) [36]. Patients were instructed that the objective was to walk as far as possible in 6 min.

### Polysomnography

All patients were invited to undergo a full-night standard polysomnography (PSG) (Resmed Nox A1). Only patients who accepted to undergo a PSG were considered for the analysis regarding PSG data. Transcutaneous carbon dioxide (PtcCO_2_) was monitored continuously overnight (SenTec Inc., Therwil, Switzerland) and was synchronized to the PSG. PSG recordings were analyzed according to the 2015 update of the American Academy of Sleep Medicine rules for scoring respiratory events in sleep [37]. Apnea was defined as the absence of airflow ≥ 10 s, hypopnea was defined as reduction of airflow ≥ 30% associated with a decrease in oxygen saturation ≥ 3% or micro-awakening. The Apnea–Hypopnea Index (AHI) was calculated as the number of apneas and hypopneas per hour of total sleep time (TST). The arousal index was defined as the number of electroencephalographic arousals per hour of TST. Periodic leg movement (PLM) was defined as four or more consecutive, involuntary leg movements during sleep, lasting 0.5–5 s with an interval of 5–90 s.

### Statistical analyses

Data are expressed as median and range for quantitative variables and as number and percentage for qualitative variables.

Comparisons between insomniac and non-insomniac patients were performed using Fisher’s exact test for categorial data. As the Kolmogorov test showed that distributions are not normal, quantititative data were analyzed using a non-parametric Wilcoxon–Mann–Whitney test to assess significance between different conditions. A Spearman test was used to study the correlation between ISI and HAD scores. For all analyses, a two-sided *p* value < 0.05 was considered significant. XLSTAT software (version 2019.1.3, Addinsoft company, Paris, France) was used to analyze and reformat data.

## Results

### Patient demographic and clinical characteristics

Fifty-nine adult patients with CF were referred to our medical center. Nine of these patients were not eligible for the study (long-term oxygen therapy (n = 1), bi-level positive airway pressure therapy (n = 5) or lung transplantation (n = 3)). Four patients refused to participate, 17 had experienced another medical condition during the previous 4 weeks and 1 woman was pregnant. Twenty-eight patients were included in the final analysis.

Median age of the 28 patients was 27 (22–34) years, including 18 men and 10 women.

Fourteen patients (50%) carried a heterozygous gene mutation (Phe508del mutation on at least one allele in 64% of cases) and 50% carried a homozygous Phe508del mutation. Eighteen patients (64%) had exocrine pancreatic insufficiency and 9 patients (32%) had diabetes. Median forced expiratory volume in one second was 2190 (1530–3667) mL, 72 (39–93) % predicted. Median consumption of alcohol, coffee and tea were 0 (0–0) g/day, 1 (0–2) cup/day and 0 (0–1) cup/day, respectively and no patient used energy drinks. Two patients (7%) were treated with anxiolytics and no patient was treated with hypnotics. Median screen time was 6 (4–8) h/day. Two patients (7%) were shift workers. Patient demographic and clinical characteristics are presented in Table 1. Twelve patients (43%) presented insomnia, defined as ISI score > 7.

**Table 1:** Patient demographic and clinical characteristics

	CF patients (n = 28)
Age (years)	27 (22–34)
Sex (M/F)	18/10
Body Mass Index (kg/m^2^)	22 (20–26)
Smoking	
Previous	4 (14%)
Current	0 (0%)
Never	24 (86%)
Pack-Years (number)	0 (0–0)
mMRC score	
≥ 1	15 (54%)
≥ 2	5 (18%)
CASA-Q score	39 (31–48)
Insomnia Severity Index	
ISI score	6 (3–14)
ISI score > 7 (insomnia)	12 (43%)
8 ≤ ISI score ≤ 14 (mild insomnia)	7 (25%)
15 ≤ ISI score ≤ 21 (moderate insomnia)	5 (18%)
22 ≤ ISI ≤ 28 (severe insomnia)	0 (0%)
Pittsburgh Sleep Quality Index	
PSQI score	4 (3–9)
PSQI score > 5	13 (46%)
Morningness-Eveningness Questionnaire	
MEQ score	54 (47–61)
Morning type	8 (29%)
Neither type	16 (57%)
Evening type	4 (14%)
Epworth Sleepiness scale	
ES score	5 (4–8)
ES score > 10	1 (4%)
Numeric Rating Scale for pain (n = 23)	1 (0–3)
Berlin scale	
Berlin score	0 (0–1)
Berlin score > 1	5 (18%)
Restless Legs Syndrome	5 (18%)
International Restless Legs Syndrome score	0 (0–0)
0 ≤ IRLS score ≤ 10 (mild RLS)	0 (0%)
11 ≤ IRLS score ≤ 20 (moderate RLS)	4 (14%)
21 ≤ IRLS score ≤ 30 (severe RLS)	1 (4%)
31 ≤ IRLS score ≤ 40 (very severe RLS)	0 (0%)
Hospital Anxiety Depression scale	
Anxiety HAD score	6 (4–9)
Anxiety HAD score > 7	10 (36%)
Anxiety HAD score > 10	4 (14%)
Depression HAD score	4 (1–7)
Depression HAD score > 7	5 (18%)
Depression HAD score > 10	2 (7%)

Data are expressed as median (interquartile range) and as number (percentage) of patients

ISI: Insomnia Severity Index, PSQI: Pittsburgh Sleep Quality Index, MEQ: Morningness-Eveningness Questionnaire, ES: Epworth Sleepiness Scale, RLS: Restless Legs Syndrome, IRLS: International Restless Legs Syndrome Scale, HAD: Hospital Anxiety and Depression scale. CFQ-R: Cystic Fibrosis Questionnaire-Revised

### Comparisons of patients with ISI score > 7 (insomnia) and patients with ISI score ≤ 7 (no insomnia)

**Fig. 1:**
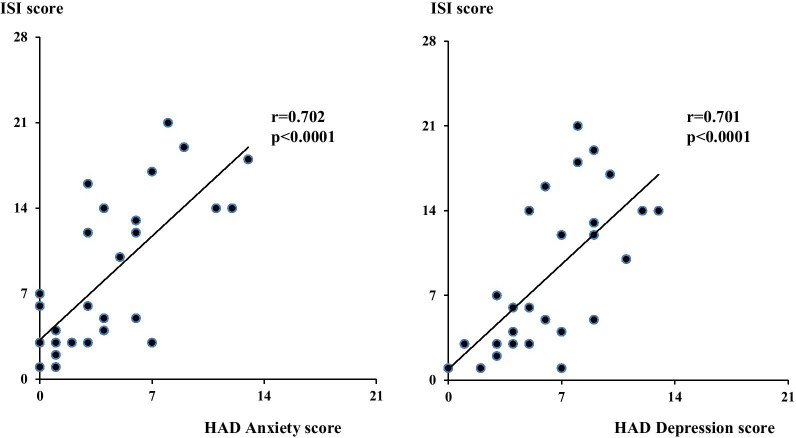
Associations between Insomnia Severity Index and Hospital Anxiety Depression scale scores. ISI: Insomnia Severity Index. HAD: Hospital Anxiety Depression scale

Patients with ISI score > 7 (insomnia) and patients with ISI score ≤ 7 (no insomnia) were compared in terms of demographic characteristics, clinical characteristics and quality of life (Table 2), respiratory assessments (Table 3) and polysomnographic data (Table 4). Compared with patients with no insomnia (ISI score < 7), patients with insomnia more frequently presented evening type (33% vs 0%, *p* = 0.02) and RLS (42% vs 0%, *p* < 0.01), more frequently experienced more severe pain (3 (1–4) vs 0 (0–1), *p* < 0.05), mMRC dyspnea ≥ 2 (42% vs 0%, *p* < 0.05) (Tables 2 and 3) and more frequently presented lower mean SpO_2_ during sleep (92 (92–94)% vs 96 (94–96)%, *p* < 0.05) (Table 4). Insomnia was also associated with an impaired quality of life in different domains of CFQ-R (physical functioning, *p* < 0.05; vitality, *p* < 0.001; emotional functioning, *p* < 0.001; health perception, *p* < 0.05; social functioning, < 0.01; body image, *p* < 0.001; role limitation, *p* < 0.001; respiratory symptoms, *p* < 0.05 and digestive symptoms, *p* < 0.05) (Table 2). We identified a strong association between insomnia and HAD anxiety (r = 0.702, *p* < 0.0001) and depression score (r = 0.701, *p* < 0.0001) (Table 2 and Fig. 1).

**Table 2:** Comparison of clinical, demographic characteristics and quality of life between patients with and without insomnia

	Patients with insomnia (ISI > 7) (n = 12)	Patients without insomnia (ISI ≤ 7) (n = 16)	*p*
Age (years)	22 (18–26)	27 (22–33)	0.62
Sex (M/F)	6 (50%)/6 (50%)	12 (75%)/4 (25%)	0.24
BMI (kg/m^2^)	21 (20–25)	22 (20–26)	0.98
Anxiolytic (n = 27)	2 (17%)	0 (0%)	0.18
Screen time (h/day)	7 (5–8)	5 (4–8)	0.74
Coffee intake (cups/day)	0 (0–2)	1 (1–2)	0.36
Tea intake (cups/day)	0 (0–1)	1 (0–1)	0.41
Alcohol intake (g/day)	0 (0–1)	0 (0–3)	0.73
Shift work	0 (0%)	2 (13%)	0.49
Insomnia Severity Index			
ISI score	14 (13–18)	3 (3–5)	** < 0.0001**
Pittsburgh Sleep Quality Index			
PSQI score	9 (7–12)	3 (3–4)	** < 0.0001**
PSQI > 5	11 (92%)	2 (13%)	** < 0.0001**
Morningness-Eveningness Questionnaire			
MEQ score	49 (41–57)	55 (52–62)	**0.03**
Morning type	2 (17%)	6 (38%)	0.40
Neither type	6 (50%)	10 (62%)	0.70
Evening type	4 (33%)	0 (0%)	**0.02**
Epworth Sleepiness scale			
ES score	5 (5–9)	5 (4–6)	0.55
ES score > 10	1 (8%)	0 (0%)	0.43
Numeric rating scale for pain (n = 22)	3 (1–4)	0 (0–1)	** < 0.05**
Berlin scale			
Berlin score	1 (0–2)	0 (0–1)	0.07
Berlin > 1	4 (33%)	1 (6%)	0.13
Restless Legs Syndrome	5 (42%)	0 (0%)	** < 0.01**
IRLS score	0 (0–13)	0 (0–0)	** < 0.05**
0 ≤ IRLS score ≤ 10 (mild RLS)	0 (0%)	0 (0%)	1.00
11 ≤ IRLS score ≤ 40 (moderate to very severe RLS)	5 (42%)	0 (0%)	** < 0.01**
Hospital Anxiety Depression scale			
Anxiety HAD score	9 (8–11)	4 (3–6)	** < 0.0001**
Anxiety HAD score > 7	9 (75%)	1 (6%)	** < 0.001**
Depression HAD score	7 (5–10)	1 (1–4)	** < 0.001**
Depression HAD score > 7	5 (42%)	0 (0%)	** < 0.01**
CFQ-R score			
Physical functioning	52 (29–79)	83 (75–92)	** < 0.05**
Vitality	42 (25–42)	67 (50–83)	** < 0.001**
Emotional functioning	64 (40–73)	93 (87–100)	** < 0.001**
Eating problems	89 (67–100)	100 (89–100)	0.20
Treatment burden	67 (44–78)	78 (56–89)	0.24
Health perception	56 (33–67)	67 (56–78)	** < 0.05**
Social functioning	61 (39–67)	83 (78–94)	** < 0.01**
Body image	56 (33–67)	78 (67–89)	** < 0.001**
Role limitations	71 (58–75)	92 (92–100)	** < 0.001**
Weight problems	67 (33–100)	67 (33–100)	0.98
Respiratory symptoms	56 (56–78)	78 (67–94)	** < 0.05**
Digestive symptoms	78 (67–89)	89 (89–100)	** < 0.05**

Data are expressed as median (interquartile range) and as number (percentage) of patients

ISI: Insomnia Severity Index, PSQI: Pittsburgh Sleep Quality Index, MEQ: Morningness-Eveningness Questionnaire, ES: Epworth Sleepiness Scale, RLS: Restless Legs Syndrome, IRLS: International Restless Legs Syndrome Scale, HAD: Hospital Anxiety and Depression scale. CFQ-R: Cystic Fibrosis Questionnaire-Revised

**Table 3:** Comparison of respiratory assessment parameters between patients with and without insomnia

	Patients with insomnia (ISI score > 7) (n = 12)	Patients without insomnia (ISI score ≤ 7) (n = 16)	*p*
CASA-Q score	51 (35–70)	34 (29–40)	** < 0.05**
Nocturnal cough	7 (58%)	6 (38%)	1.00
mMRC score			
≥ 1	7 (58%)	8 (50%)	0.72
≥ 2	5 (42%)	0 (0%)	** < 0.05**
Nasal obstruction	6 (50%)	2 (13%)	0.09
Pulmonary exacerbations	1 (0–2)	1 (0–2)	0.64
Number per year			
Number over last 3 months	1 (0–1)	0 (0–1)	0.78
At least 1 exacerbation in the past year	6 (50%)	10 (62%)	0.70
At least 1 exacerbation in the past 3 months	6 (50%)	7 (44%)	1.00
At least 1 antibiotic use in the past year	4 (33%)	2 (13%)	0.35
Bacterial colonization of the airways (n = 27)			
*Pseudomonas aeruginosa*	3 (25%)	11(69%)	0.24
*Burkholderia cepacia*	0 (0%)	0 (0%)	1.00
*Stenotrophomonas maltophilia*	1 (8%)	0 (0%)	0.44
*Achromobacter xylosoxidans*	0 (0%)	0 (0%)	1.00
Pulmonary function tests			
FEV_1_ (mL)	2885 (1470–3490)	2065 (1540–4030)	0.93
FEV_1_ (% predicted)	74 (40–108)	66 (38–89)	0.42
FVC (mL)	3945 (2720–4435)	3530 (2680–5100)	1.00
FVC (% predicted)	97 (65–113)	86 (65–97)	0.22
FEV_1_/FVC	64 (55–82)	69 (53–74)	0.70
6 MWT (n = 16)			
SpO_2_ min (%)	93 (91–96)	96 (93–97)	0.25
Distance (m)	496 (425–584)	581 (505–630)	0.21

Data are expressed as median (interquartile range) and as number (percentage) of patients

CASA-Q: Cough And Sputum Assessment, mMRC: modified Medical Research Council, FEV_1_: forced expiratory volume in 1 s, FVC: forced vital capacity, 6 MWT: 6-min walking test, SpO_2_: pulse oxygen saturation

**Table 4:** Comparison of polysomnographic data between patients with and without insomnia

	Patients with insomnia (ISI score > 7) (n = 8)	Patients without insomnia (ISI score ≤ 7) (n = 6)	*P*
Sleep Latency (min)	26 (16–29)	19 (11–25)	0.57
Sleep latency > 45 min	1 (13%)	1 (17%)	1.00
Total Sleep Time (min)	415 (343–500)	453 (441–526)	0.35
N1 (min)	16 (8–21)	10 (8–15)	0.57
N1 (% TST)	4 (2–5)	2 (2–3)	0.32
N2 (min)	242 (182–274)	195 (183–234)	0.57
N2 (% TST)	51 (44–57)	45 (40–52)	0.29
N3 (min)	80 (72–118)	124 (95–145)	0.16
N3 (% TST)	21 (15–26)	26 (21–32)	0.42
REM (min)	87 (65–112)	119 (112–164)	0.13
REM (% TST)	22 (18–23)	27 (25–30)	0.05
Sleep Efficiency (%)	88 (86–92)	94 (88–96)	0.18
Arousal index (/h)	11 (6–14)	8 (5–12)	0.35
Wake after sleep onset (min)	60 (35–109)	37 (28–81)	0.66
PLM (/h)	0 (0–0)	0 (0–0)	1.00
PLM > 15/h	0 (0%)	0 (0%)	1.00
Apnea–Hypopnea Index (/h)	0 (0–2)	1 (0–4)	0.85
Mean SpO_2_ during sleep (%)	92 (92–94)	96 (94–96)	** < 0.05**
% SpO_2_ < 90% asleep	0 (0–3)	0 (0–0)	0.53
Oxygen Desaturation Index (3%) (/h)	3 (2–7)	4 (3–6)	0.45
Mean PtcCO_2_ during sleep (mmHg)	45 (41–46)	46 (43–47)	0.25
Max PtcCO_2_ during sleep (mmHg)	47 (44–50)	50 (47–51)	0.59
% PtcCO_2_ > 50 mmHg	0 (0–2)	0 (0–2)	1.00

Data are expressed as median (interquartile range) and as number (percentage) of patients

REM: rapid eye movement sleep, PLM: periodic leg movements, SpO_2_: pulse oxygen saturation, PtcCO_2_: transcutaneous carbon dioxide pressure

Polysomnography was performed in 14 patients. Patients in whom polysomnography was performed had similar characteristics to those in whom PSG was not performed, except for ISI score (13 (5–17) for patients with PSG vs 6 (2–9) for patients without PSG, *p* = 0.022) and HAD depression score (6 (3–9) for patients with PSG vs 2 (1–5) for patients without PSG, *p* = 0.029).

Two patients (14%) had sleep latency > 45 min. Only one patient (7%) had sleep efficiency < 80%, total sleep time < 360 min, AHI ≥ 15/h and severe nocturnal hypoxemia (mean SpO_2_ during sleep: 87%, with SpO_2_ < 90% during 77% of sleep time). Thirteen patients (93%) had a mean SpO_2_ during sleep > 92%. No patient had periodic limb movements.

## Discussion

This study specifically assessed insomnia-related factors in adult CF patients. It highlighted a high prevalence of insomnia in adult CF patients with 43% of patients exhibiting an ISI score > 7 and, in particular, showed that insomnia is strongly associated with anxiety/depression symptoms in this population.

Several screening tools can be used to diagnose insomnia. A recent meta-analysis compared the three scales most commonly used to screen for insomnia: PSQI, Athens Insomnia Scale (AIS) and ISI [23] and identified comparable diagnostic properties, with AIS and ISI providing better results in terms of specificity than PSQI. Of note, PSQI does not directly assess insomnia symptoms, but rather evaluates a broad range of sleep domains affecting sleep quality. AIS and ISI were developed according to standard insomnia diagnostic criteria. The AIS was designed to quantify sleep difficulties based on the International Classification of Diseases and Related Health Problems-10 (ICD-10). The ISI captures the diagnostic criteria for insomnia defined in Diagnostic and Statistical Manual of Mental Disorders-IV (DSM-IV) and the International Classification of Sleep Disorders (ICSD). In addition, ISI is easy to use and not time-consuming in clinical practice. As expected, the presence of insomnia according to ISI was associated with a higher PSQI score in our study.

Our study showed a high prevalence of insomnia in clinically stable adult CF patients (43%), which is closely comparable to that a previous study in CF young adults, using AIS (41.7%) [12]. For comparison, the prevalence of insomnia in healthy young adults (25–34 years old) is about 18% in France, according to a large epidemiological study conducted in 2001 [38]. Forty-six percent of patients in our study also had poor sleep quality (i.e., PSQI score > 5). These results are similar to those of several studies conducted in CF adults that reported impaired sleep quality according to PSQI in 37 to 66% of patients [8, 10, 11, 15].

RLS was significantly more common in insomniac patients in our study. A recent study has shown that about one-third of adult CF patients experience RLS [16]. The prevalence of RLS was lower in our study population (18%), but the presence of RLS was systematically associated with an ISI score > 7, suggesting that RLS may have a negative impact on sleep quality. Periodic limb movements were not observed in patients who underwent full-night polysomnography, but they may be absent during PSG in a considerable proportion of patients.

Pain [15], with a mean score of "mild" in these stable patients, and dyspnea were the two symptoms more frequently associated with insomnia in our study.

Apart from mean SpO_2_ during sleep [9], we did not find any associations between insomnia, as assessed by ISI, and physiological variables describing the severity of respiratory disease, probably due to the less marked respiratory impairment in our study compared to the study by Milross et al. [10] (mean FEV_1_ 72 (39–93)% *vs* mean FEV_1_ 36 ± 12%). Decreased mean SpO_2_ during sleep in insomniac patients could explained the fact that dyspnea is more severe in this group.

The main factor associated with insomnia in our study was higher HAD anxiety and depression scores. It was already shown that reduced sleep quality and daytime sleepiness were associated with poorer mood in children and young adults with CF [6, 12]. Available data suggest that, as in other chronic diseases, symptoms of anxiety and depression are common features in CF patients. In a study carried out on 4,739 adult CF patients across nine countries in Europe and the USA, symptoms of anxiety and depression were found in 32% and 19% of patients, respectively (36% had an HAD anxiety score > 7, 18% had an HAD depression score > 7 in our study). Overall, anxiety and depression scores were 2- to threefold higher than those of community samples [39]. Although anxiety and depression are known to be a cause of insomnia [40], a causal relationship cannot be established between insomnia and anxiety/depression, but the presence of insomnia symptoms should prompt physicians to investigate the presence of and treat anxiety/depression symptoms and vice versa.

Quality of life was more severely impaired in patients with insomnia, especially for the categories concerning physical functioning, vitality, emotional state, health perception, social functioning, body image, role limitations and respiratory and digestive symptoms. These results are consistent with those of a study reported by Bouka et al. [11], in which lower sleep quality was related to vitality, emotional functioning, social, role, eating disturbances and digestive symptoms. It is noteworthy that these symptoms may also be an expression of anxiety and/or depression.

Total sleep time and sleep architecture, analyzed by PSG, were generally normal in patients with and without insomnia. Total sleep time on PSG was slightly longer in patients without insomnia than in patients with insomnia and the time spent awake after sleep onset was slightly, but not significantly shorter, probably due to the lack of power of the study.

This study has several limitations. First, it was a single-center study comprising a small number of patients, which may limit the generalizability of the results. Given the low number of patients, a comparative analysis by gender was not possible [41]. It would also have been interesting to assess insomnia by actimetry over several days to obtain objectives measurements on activity and rest periods. Despite these limitations, this pragmatic study presents a number of important strengths, including assessment of the relationships in CF adult patients between insomnia, assessed by ISI, and multidimensional parameters including demographic and clinical characteristics, and a global respiratory assessment (symptoms, airway colonization, PFTs, 6MWT, polysomnography) with minimal exclusion criteria.

## Conclusion

This study shows that insomnia is a common complaint in CF adults and is associated with many factors: more severe dyspnea, pain, RLS, lower SpO_2_ during sleep and especially anxiety/depression symptoms. The strong association between insomnia, impaired quality of life and higher HAD score should prompt physicians to be particularly attentive to the management of anxiety and depression in adult CF patients with insomnia.

## Data Availability

The datasets used and/or analysed during the current study are available from the corresponding author on reasonable request.

## Abbreviations

CF: Cystic fibrosis
PSQI: Pittsburgh Sleep Quality Index
ISI: Insomnia Severity Index
BMI: Body Mass Index
CASA-Q: Cough And Sputum Assessment Questionnaire
mMRC: Modified Medical Research Council dyspnea scale
NRS: Numeric Rating Scale for pain
ES: Epworth Sleepiness Scale
MEQ: Morningness-Eveningness Questionnaire
HAD: Hospital Anxiety Depression scale
IRLS: International Restless Legs Syndrome scale
CFQR 14+: Cystic Fibrosis Questionnaire-Revised
PFTs: Pulmonary Function Tests
6MWT: 6-Min walking test
AHI: Apnea Hypopnea Index
TST: Total Sleep Time
PLM: Periodic Leg Movements
AIS: Athens Insomnia Scale
